# Eccentric Exercise Reduces Upper Trapezius Muscle Stiffness Assessed by Shear Wave Elastography and Myotonometry

**DOI:** 10.3389/fbioe.2020.00928

**Published:** 2020-08-05

**Authors:** Aleksandra Kisilewicz, Pascal Madeleine, Zofia Ignasiak, Bogdan Ciszek, Adam Kawczynski, Ryan Godsk Larsen

**Affiliations:** ^1^Department of Paralympics Sports, University School of Physical Education, Wrocław, Poland; ^2^Sport Sciences – Performance and Technology, Department of Health Science and Technology, Aalborg University, Aalborg, Denmark; ^3^Department of Biostructure, University School of Physical Education, Wrocław, Poland; ^4^Department of Descriptive and Clinical Anatomy, Medical University of Warsaw, Warsaw, Poland

**Keywords:** eccentric exercise, trapezius muscle, muscle stiffness, ultrasonography, myotonometry

## Abstract

In this study, we tested the hypotheses that unaccustomed eccentric exercise (ECC) would reduce the elastic modulus and dynamic stiffness of the upper trapezius muscle and that these changes would correlate with increases in muscle thickness, reflecting muscle edema. Shear wave elastography was used to measure elastic modulus, dynamic stiffness was assessed using myotonometry, and muscle thickness was measured using ultrasonography. All measurements were performed at four locations over the upper trapezius before and 24 h after a single bout of ECC. Fourteen healthy participants (11 males and 3 females; 23.2 ± 3.0 years; height 175.1 ± 10.4 cm; body mass 73.8 ± 11.3 kg) took part in the study. Overall, ECC resulted in decreased elastic modulus (from 45.8 ± 1.6 to 39.4 ± 1.2 kPa, *p* < 0.01) and dynamic muscle stiffness (from 369.0 ± 7.3 to 302.6 ± 6.0 N/m, *p* < 0.01). Additionally, ECC resulted in increased muscle thickness (from 6.9 ± 0.4 to 7.3 ± 0.4 mm, *p* < 0.01). Spatial changes (across the four locations) were found for elastic modulus, stiffness and thickness. No significant correlations were found between changes in measures of muscle stiffness, or between changes in stiffness and changes in thickness. In conclusion, the present pilot study showed that ECC altered biomechanical muscle properties, reflected by decreased elastic modulus and dynamic muscle stiffness 24 h after ECC.

## Introduction

In a biomechanical context, stiffness refers to the resistance of tissue during passive stretching which depends on the type of external force applied and the deformation of the structure caused by this force (Baumgart, 2000). Increased level of stiffness has been related to a higher risk of strain and stress injuries (Pruyn et al., 2012). A recent study on overhead athletes have shown that neck and shoulder pain is related to higher stiffness of upper trapezius (UT) muscle (Leong et al., 2016). More specifically, the shoulder girdle and the trapezius are of special interest in both sport and occupational perspectives when one considers the high prevalence of neck-shoulder disorders and their socioeconomic burden (Picavet and Hazes, 2003).

Unaccustomed eccentric muscle contractions place high strain on the muscle-tendon complex resulting in neuromuscular functional impairments, muscle damage and finally delayed onset muscle soreness (DOMS) (Guilhem et al., 2010). According to the literature, muscle soreness peaks between 12- and 36-h after exercise (Nosaka et al., 2002). Soreness sensation is related to the inflammation process due to efflux of substances from the damaged tissue to the extracellular space that sensitize free nerve endings and activate groups III and IV pain receptors (Zainuddin et al., 2005). Eccentric exercise (ECC) has also been reported to increase muscle stiffness (Hoang et al., 2007). It has been suggested that post-exercise stiffness is caused by muscle edema related to fiber damage. On the other hand, change in muscle stiffness precedes any evidence of swelling (Proske and Morgan, 2001). Particularly, muscle edema occurs subsequent to the inflammatory response and prior to the first symptoms of DOMS (Peake et al., 2005). Moreover, several studies show no correlation between exercise–induced change in muscle stiffness and typical indicators of muscle damage, such as edema and soreness (Lacourpaille et al., 2014; Yanagisawa et al., 2015). Therefore, there is a need for further investigating the DOMS related changes in mechanical properties of skeletal muscles.

The assessment of biomechanical properties of the musculoskeletal system is challenging because striated muscle is an anisotropic and viscoelastic complex composed of both active and passive structures (Gennisson et al., 2010). However, relatively new methods based on shear wave elastography (SWE) and myotonometry have been reported to provide reliable assessments of biomechanical properties of individual muscles (Lacourpaille et al., 2012; Kawczynski et al., 2018). Ultrasonic SWE provides real-time, direct measurement of muscle elastic modulus. The SWE estimates shear elastic modulus linearly related to Young’s modulus, as a substitute for passive muscle stiffness (Lacourpaille et al., 2017; Xie et al., 2019; Zhang et al., 2019). In recent years, the application of SWE for muscle tissue evaluation has increased rapidly, with good-to-excellent intra-observer, inter-observer, and inter-day reliability of SWE for elastic modulus measurements (Tas et al., 2017). The SWE methodology has been proposed to non-invasively quantify exercise-induced muscle damage, with relevance for clinical evaluation of athletes (Lacourpaille et al., 2017). Myotonometry is an alternative method that quantifies soft tissue viscoelastic properties, including muscle stiffness. Importantly, myotonometry is less expensive than SWE, hand-held and easy to use, with application for use in sport fields and every-day evaluation of training or treatment. Similarly, to SWE, myotonometry has been successfully employed in the evaluation of viscoelastic muscle parameters (Gervasi et al., 2017), showing good validity and high reliability for e.g., the trapezius muscle (Viir et al., 2011; Kawczynski et al., 2018). Although both SWE and myotonometry operate using the principle of Young’s modulus, the depth of measurements varies, as SWE provide measurements of elastic modulus (i.e., passive muscle stiffness) from deeper structures, while myotonometry measures dynamic muscle stiffness superficially (Kelly et al., 2018). Further, these measurements made at defined locations enabled inverse-distance-based interpolation to generate topographical maps of the viscoelastic properties (Kawczynski et al., 2018; Heredia-Rizo et al., 2020).

To date, no prior study has compared the spatial changes in biomechanical properties of skeletal muscle, using both SWE and myotonometry, following unaccustomed eccentric muscle contractions. Therefore, the present study was designed to quantify the spatial changes of UT elastic modulus, muscle stiffness and thickness, 24 h after unaccustomed ECC of UT. Based on our previous work (Kawczynski et al., 2018), we hypothesized that ECC would reduce the elastic modulus and stiffness of UT and that these changes would correlate with increases in muscle thickness, reflecting exercise-induced muscle edema.

## Materials and Methods

### Participants

Fourteen participants (11 males and 3 females; aged 23.2 ± 3.0 years; height 175.1 ± 10.4 cm; body mass 73.8 ± 11.3 kg) volunteered to take part in this pilot study. All participants were recruited form mixed student population, with no professional training experience in disciplines involving overhead actions or occupations with significant involvement of the upper limbs. All participants were right-handed and maintained normal daily activity during the course of the study. The inclusion criteria were: no pain in the shoulder region prior to the experiment, no history of neck or shoulder disorders, and no strength training in the past month. The study was reviewed and approved by the Research Ethics Committee of North Denmark (N-2016-0023). The participants provided their written informed consent to participate in this study.

### Study Design

All measurements were carried out by the same investigator, trained in muscle ultrasonography and myotonometry. The study was performed in the spring season, with an average indoor temperature of 25°C. The Young’s modulus and stiffness of UT muscle on the dominant side, reported by each participant prior the experiment. During the experiment, participants sat on a chair with the back supported in an upright position. Forearms were supported on the table, and the head was facing forward. The skin overlying UT muscle was exposed for testing. Thickness measurements were acquired first followed by myotonometry and SWE measurements. The measurements were performed twice: before the ECC protocol, and 24 (±1) h post ECC.

**FIGURE 1:**
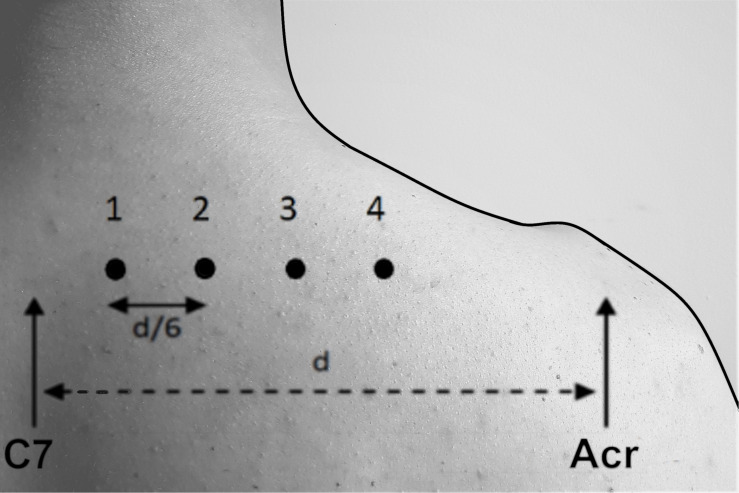
Location of the four measurement points marked on the skin overlying the upper trapezius muscle for the assessment of muscle dynamic stiffness measured by myotonometry, elastic modulus measured by shear-wave elastography and thickness measured by ultrasonography C7, 7th cervical vertebrae spinous process; Acr., acromion; “d”, the distance between C7 spinous process and acromion.

Myotonometry, SWE and muscle thickness measurements were performed on the same four points marked on the skin overlying the UT muscle (Figure 1). To properly locate four points of measurement, the spinous process of the C7 vertebra and the angle of acromion were identified by palpation. The distance between C7 spinous process and acromion: “d,” was used to calculate the between-points distance (mean 21.8 ± 1.8 cm). Adjacent points were separated by 1/6 (mean 3.6 ± 0.3 cm) of “d” distance (Kawczynski et al., 2018). The B-mode ultrasound images were used to locate the greatest muscle thickness and confirm correct points location. The probe of the MyotonPRO device was positioned directly over each point. The device would only start the measurement when the investigator applied the appropriate pre-compression force and the probe was placed perpendicular to the surface of the skin. For SWE measurements, the transducer was oriented in parallel to the UT muscle fibers, with the center positioned over the skin mark. During data acquisition, the transducer was held stationary for 10 s during which the SWE sonogram was recorded with minimal pressure applied on the skin (Kot et al., 2012). A rectangular region of interest (ROI) selected to determine the shear elastic modulus of the UT muscle was defined by the thickness of the muscle belly, excluding aponeurosis and unfilled regions within the elasticity map (Ates et al., 2015). Due to differences in anatomy, there were slight variation in the ROI used across participants. All ultrasonic images were imported to a personal laptop and given specific codes in order to blind the identity of the data. This process was done by an investigator who was not involved in data acquisition or analyses. To obtain highest reliability of measurements we used an average of three SWE elasticity measurements of elastic modulus (Kelly et al., 2018), and a MyotonPRO Multiscan mode of five continuous measurements of dynamic muscle stiffness (Vain and Kums, 2002).

Three LOGIQ longitudinal view images of UT muscle belly at C7 level were performed before and 24 h post ECC. Thickness of UT was determined using MicroDicom viewer software (MicroDicom DICOM Viewer, Bulgaria). Specifically, four parallel straight lines were drawn between the superficial and deep fascia of UT muscle belly. Averages from three images for each of four measurement points were calculated and used for further analyses. The muscle stiffness, SWE elastic modulus and thickness maps were created using the averaged values for each of the assessed points (Figure 3). The interpolation was done using an inverse distance weighted interpolation to obtain a 3D graphical representation (Binderup et al., 2010b).

In addition to measurements of UT dynamic stiffness, elastic modulus, and thickness, measurements of range of movement (ROM) for shoulder elevation, maximum voluntary contraction (MVC) in isometric condition, and soreness intensity was obtained before and 24 h post ECC. Each participant reported individual soreness level, using a 10-cm standardized visual analog scale where 0 indicated “no soreness” and 10 “maximal soreness intensity.”

### Ultrasound Measurements

An ultrasound system (LOGIQ S8, General Electric, Norwalk, CT, United States) was used for acquiring images (B-mode, 8.5–10.0 MHz) of the UT muscle. For the assessment of UT muscle thickness, the LOGIQ longitudinal view feature was used to acquire an image over the full length of the UT muscle at the C7 level of the cervical spine. Specifically, a linear transducer (9L) was positioned over the right UT, parallel to the arrangement of muscle fibers for measurements of thickness and SWE. The orientation of the transducer longitudinal to muscle fibers is required to achieve accurate and reliable measurements (Gennisson et al., 2010). The velocity of the shear wave propagation tracked by a pulse-echo ultrasound, provides a characterization of elastic properties as velocity of shear waves increase with increase in passive muscle stiffness (Eby et al., 2013). Assuming a linear and elastic behavior, the shear elastic modulus (kPa) is a function of shear velocity (Vs) as follows: *E* = ρVs^2^, where ρ is the density of muscle (1000 kg/m^3^) (Ates et al., 2015).

### Myotonometry Measurements

The MyotonPRO (Myoton AS, Myoton Ltd., Estonia) is a non-invasive, portable device using superficial mechanical deformation for assessment of biomechanical characteristics of soft tissues (Aird et al., 2012). The device has been used to measure dynamic stiffness (N/m) of trapezius muscles, quantified with the damped oscillation method (Viir et al., 2011). Dynamic stiffness is defined as the resistance of a soft tissue to a contraction or an external force that deforms its initial shape (Schneider et al., 2015). The probe of the device was placed perpendicular to the surface of the skin overlying the UT, and applied slight pressure with a short mechanical impulse (0.4 N for 15 ms), with a constant pre-compression force of 0.18 N. The mechanical impulse generated damped oscillations within the muscle soft tissue, recorded by an accelerometer. The acceleration signal is processed to provide an oscillation curve from which the dynamic stiffness is calculated as follows: the period T, is defined as the time elapsed between the first two adjacent acceleration peaks following the mechanical impulse; the oscillation frequency *f* is calculated from the equation: *f* = 1/T; the angular frequency ω (ω = 2π*f*), is related to stiffness K and mass m, and allows for calculating stiffness: *K* = ω^2^m, substituting into: *K* = 4π^2^*f*^2^m (Sohirad et al., 2017).

### Eccentric Exercise Protocol

A dynamic shoulder dynamometer (Aalborg University, Aalborg, Denmark) was used to induce DOMS in the UT muscle (Madeleine et al., 2006). The dynamic shoulder dynamometer consists of an actuator, a load cell, a control unit, a cylinder, a shoulder contact pad, and an adjustable seat fixed on a stainless-steel frame. The ECC protocol consisted of 50 ECC performed in five bouts of 10 contractions at 100% level of maximum voluntary contraction (MVC), separated by a 2-min rest (Kawczynski et al., 2012). Due to the one-sided design of the dynamometer, the ECC was performed only with the right (dominant) UT muscle.

Before exercising, the ROM for shoulder elevation was assessed. All participants were asked to elevate their right shoulder to their highest position and then lower it as much as possible. Both positions were measured and recorded by the dynamometer. The ROM was calculated as the difference between the highest and lowest position. In the eccentric mode, the dynamometer produces a constant vertical downward force at a given level of MVC. Three trials were performed to determine the MVC in neutral position, i.e., the maximal shoulder elevation force under isometric conditions for 3 s, separated by 2 min rest. The contact point between the dynamometer and shoulder was approximately 3 cm medial to the acromion. The contact pad of the shoulder dynamometer was padded to avoid inhibition of force exertion because of pressure pain (Kawczynski et al., 2007). While exercising, participants were instructed to counteract the vertical force, generated by the dynamometer, along the shoulder ROM. All participants wore a corset during the exercise to prevent lateral bending.

### Statistical Analysis

The sample size was calculated using G^∗^Power software (Kiel University, Kiel, Germany) with an expected “medium” effect size (*f*^2^ = 0.25) for changes over time, α level of 0.05, power (1-β) of 0.9, and correlation for repeated measures of 0.6 (Faul et al., 2007; Kawczynski et al., 2018). Sample size calculations revealed that 11 participants were required. Accounting for potential dropouts, 14 participants were recruited. The dynamic stiffness measured with myotonometry, elastic modulus assessed by SWE, and muscle thickness were introduced in a two-way repeated measure analysis of variance (RM-ANOVA). Time (before and 24 h post ECC) and points (1–4) marked on the skin overlying UT muscle were introduced as within-subject factors. Soreness intensity, ROM, and MVC were analyzed using a 1-way RM-ANOVA with time (before and 24 h post ECC) as within-subject factor. Normality of the data distribution was tested by Shapiro–Wilk tests. If there was an interaction between variables, Bonferroni adjustment for multiple comparisons was used for *post hoc* tests. Pearson’s product-moment correlation was used to assess the relationship between relative changes in stiffness measured by MyotonPRO and elastic modulus determined by SWE, and the association between relative changes in muscle stiffness and muscle thickness. These analyses were done for each of the four points of measurement. For all statistical tests, *p*-value < 0.05 was considered significant. Data are presented as mean ± SD in the text and the figures. All statistical analyzes were performed using SPSS 22.0 software (IBM SPSS Inc., Armonk, NY, United States).

## Results

### Myotonometry

**FIGURE 2:**
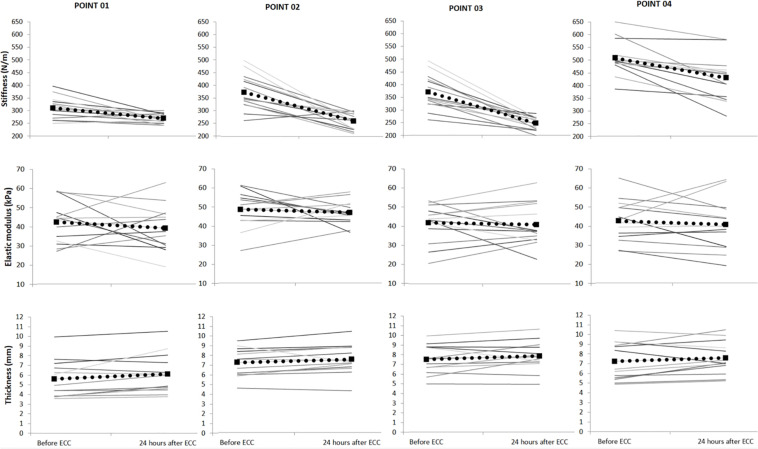
Mean (SD) muscle dynamic stiffness measured by myotonometry, elastic modulus measured by shear-wave elastography and muscle thickness measured by ultrasonography (*N* = 14), before and 24 h after eccentric exercise (ECC). Each *gray line* represents dynamic stiffness, elastic modulus and thickness variability for each of the participants. The *dotted line* represents the group mean.

There was a time-by-point interaction for muscle dynamic stiffness (*F*_1_,_13_ = 7.1; *p* = 0.022). *Post hoc* analyses revealed that muscle dynamic stiffness decreased from before (369.0 ± 7.3 N/m) to 24 h post ECC (302.6 ± 6.0 N/m) for all four points of measurement (*p* < 0.001) (Figure 2). There was a main effect of point for muscle dynamic stiffness (*F*_1_,_13_ = 62.3; *p* < 0.001). *Post hoc* analyses showed that muscle dynamic stiffness was higher in point 4 compared with all other points (all *p* < 0.001).

### Ultrasonography

There was no time-by-point interaction for SWE muscle elastic modulus (*F*_1_,_13_ = 0.1, *p* = 0.943). However, there was a main effect of time for SWE muscle elastic modulus (*F*_1_,_13_ = 12.4; *p* = 0.005), such that muscle elastic modulus decreased from before (45.8 ± 1.6 kPa) to 24 h post ECC (39.4 ± 1.2 kPa) (Figure 2). There was also a main effect of point for muscle elastic modulus (*F*_1_,_13_ = 5.4; *p* = 0.022), such that muscle elastic modulus was higher in point 1 compared with point 2 (*p* < 0.05). There was no time–by-point interaction for muscle thickness (*F*_1_,_13_ = 0.1, *p* = 0.97). However, there was a main effect of time for muscle thickness (*F*_1_,_13_ = 7.9; *p* < 0.001), such that muscle thickness increased from before (6.9 ± 0.4 mm) to 24 h post ECC (7.3 ± 0.4 mm) (Figure 2). There was also a main effect of point (*F*_1_,_13_ = 7.7; *p* < 0.001) such that muscle thickness was lower for point 1 compared with points 2 and 3 (*p* < 0.05).

### Soreness Intensity, Maximal Voluntary Contraction, Range of Motion

Soreness intensity increased from before (0.0 ± 0.0) to 24 h after ECC (4.6 ± 1.4; *p* < 0.001). There was no significant change in MVC from before (622.4 ± 243.0 N) to 24 h post ECC (521.7 ± 239.2 N; *p* = 0.10). Similarly, there was no change in shoulder elevation ROM from before (67.9 ± 16.4 mm) to 24 h post ECC (66.0 ± 9.2 mm; *p* = 0.70).

### Correlations Among Stiffness Measures and Between Stiffness and Thickness

Pearson’s product-moment correlations showed no correlation between relative changes in stiffness measured by the MyotonPRO and SWE (Table 1). Also, there were no correlations between relative changes in SWE and muscle thickness, or relative changes in myotonometry and muscle thickness (Table 1).

**TABLE 1:** Correlation coefficients between relative changes in upper trapezius muscle dynamic stiffness measured by myotonometry, elastic modulus measured by shear-wave elastography and muscle thickness measured by ultrasonography, respectively.

Point	Mean ± SD		Mean ± SD		Mean ± SD		Correlation coefficients					
	Dynamic stiffness (N/m)		Elastic modulus (kPa)		Thickness (mm)		Δ Dynamic stiffness (%) / Δ elastic modulus (%)		Δ Dynamic stiffness (%) / Δ thickness (%)		Δ Elastic modulus (%) / Δ thickness (%)	
						
	Before	24 h after ECC	Before	24 h after ECC	Before	24 h after ECC	*R*	*p*	*R*	*p*	*R*	*p*
1	309.29 ± 42.83	268.64 ± 17.72	41.96 ± 10.67	38.82 ± 11.40	5.60 ± 1.84	6.11 ± 1.96	0.08	0.78	−0.08	0.79	−0.26	0.38
2	327.07 ± 63.95	260.64 ± 31.55	47.83 ± 9.13	46.19 ± 5.99	7.19 ± 1.38	7.50 ± 1.46	−0.14	0.64	0.42	0.14	0.04	0.90
3	373.71 ± 68.79	248.64 ± 25.38	41.69 ± 9.61	40.74 ± 10.84	7.53 ± 1.42	7.87 ± 1.44	−0.25	0.39	0.14	0.63	0.08	0.79
4	500.43 ± 65.22	424.36 ± 83.24	42.47 ± 10.85	40.60 ± 13.11	7.33 ± 1.88	7.67 ± 1.63	0.50	0.07	−0.44	0.11	−0.10	0.73

## Discussion

In this pilot study, we showed a decrease in both dynamic muscle stiffness assessed by myotonometry and the elastic modulus measured by SWE at 24 h after ECC. Accompanying these changes, UT muscle thickness was increased at 24 h after ECC, indicating muscle edema (Figure 2). Our results confirmed the hypothesis concerning spatial changes in UT elastic modulus, stiffness and thickness 24 h after ECC. However, we found no significant correlations between decreases in muscle stiffness measured by SWE and myotonometry, or between decreases in stiffness and increases in thickness.

### Effect of ECC on Muscle Stiffness

In line with recent studies, we found a reduction in elastic modulus and dynamic stiffness (Andonian et al., 2016; Kawczynski et al., 2018; Xu et al., 2019). Kawczynski et al. (2018) observed a decrease in UT muscle dynamic stiffness at 24 h after a single bout of ECC, using the same exercise protocol. They also investigated test-retest reliability of MyotonPRO device, obtaining ICC (95% CI) values from 0.59 to 0.96, with standard error of measurement (±SD) of 18.5 (±7.3) and minimal detectable change (±SD) of 51.2 (±20.3), respectively. Andonian et al. (2016) demonstrated that prolonged, low-intensity, and mainly ECC induced a decrease in quadriceps muscle stiffness quantified by SWE. They showed good-to-high intra-session reliability (ICC = 0.88–0.92) of the shear modulus, with standard error of measurement from 0.12 to 0.20. Although the ECC protocol differed from our study, we used the same measurement conditions, such that the muscle was investigated in slack position (i.e., no muscle tension). As proposed by Andonian et al. (2016), the measurement conditions (slack versus stretched) could have contributed to post-exercise changes in elastic modulus. Consistent with this interpretation, Lacourpaille et al. (2017) suggested that the effect of ECC on muscle stiffness is muscle length dependent. Another study by Lacourpaille et al. (2014) showed a significant increase in elastic modulus of the elbow extensors, but only when the investigated muscles were in a stretched position. This could be explained by perturbations of calcium homeostasis, as muscle fiber sensitivity to Ca^2+^ increases with muscle elongation. Therefore, measurements performed in slack position versus stretched position could possibly contribute to discrepancies between studies (Green et al., 2012; Lacourpaille et al., 2017; Xie et al., 2019). However, the reduction in elastic modulus and dynamic stiffness stands inconsistent with the general idea of an increase in muscle stiffness after a single bout of ECC (Green et al., 2012; Heredia-Rizo et al., 2020). Green et al. (2012) reported a 21% increase in gastrocnemius medialis muscle elastic modulus, but no significant changes in soleus muscle after a single bout of ECC. Nevertheless, they used a different measurement technique (i.e., magnetic resonance elastography). Furthermore, as proposed by the authors, differences in the articular anatomy and fiber composition of various muscles may contribute to muscle dependent changes in stiffness (Green et al., 2012). In line with this idea, Xu et al. (2019) showed a significant decrease in elastic modulus of the vastus lateralis, but not the other quadriceps muscle heads after a single bout of 75 knee flexions performed in a dynamometer.

### The Relationship Between Post-exercise Changes of Muscle Stiffness and Thickness and Correlations Among Stiffness Measures

By definition, the shear elastic modulus is a function of muscle tissue density and shear wave propagation speed (Ates et al., 2015). Thereby, exercise-induced changes in muscle tissue density could affect elastic modulus after ECC. It is known that the rate of collagen turnover increases with exercise (Hjorth et al., 2015). The elevated collagen turnover increases the density of extracellular matrix (ECM) within muscle fibers, causing a highly negative interstitial fluid pressure, and subsequently a muscle edema (Kjaer, 2004). Likewise, according to Hyldahl and Hubal (2014) and Mackey et al. (2004), the changes in muscle biomechanical properties following unaccustomed ECC are most likely related to the remodeling of the ECM. However, it is surprising how little is known about ECM remodeling, despite its’ important role in myofibrillar adaptation to exercise (Kjaer, 2004). As proposed by Andonian et al. (2016), an increase in muscle stiffness after ECC could be counterbalanced by changes in extracellular water volume. As suggested by Madeleine et al. (2018), changes in the muscle physical milieu at 24 h after ECC could be related to increased muscle thickness. However, we observed no significant correlation between changes in muscle stiffness and thickness. Similarly, there was no correlation among stiffness measures, although both SWE and myotonometry showed a decrease in UT muscle stiffness 24 h after ECC. In agreement with our results, Akagi and Kusama (2015) showed no correlation between stiffness measured by SWE and a mechanical stiffness meter in the assessment of neck and shoulder muscles. On the contrary, Kelly et al. (2018) observed a significant positive correlation between myotonometry and SWE in the evaluation of infraspinatus, erector spinae and gastrocnemius muscles stiffness at rest. However, none of these studies assessed the correlation between changes in stiffness parameters after ECC. Due to the small sample size in this pilot study, there is a need for further research with larger sample sizes to investigate the relationship between muscle stiffness assessed by SWE and myotonometry.

### Post-exercise Changes of Soreness Intensity, Muscle Force, Range of Shoulder Elevation and the Spatial Heterogeneity in UT Muscle Stiffness and Thickness

**FIGURE 3:**
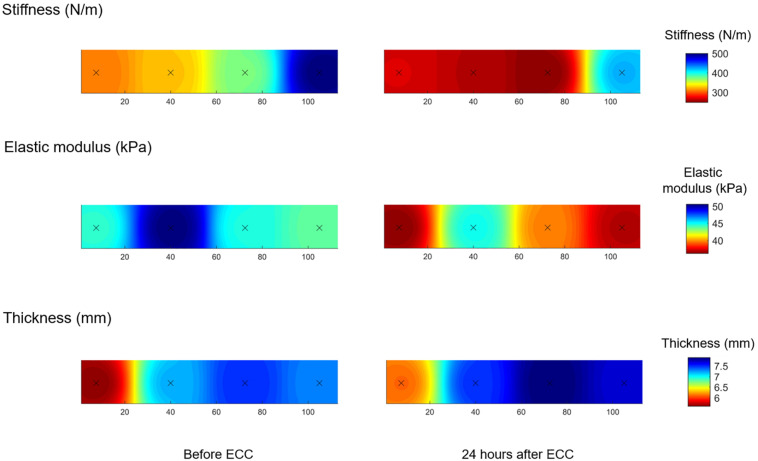
Average topographical maps of the upper trapezius muscle dynamic stiffness measured by myotonometry, elastic modulus measured by shear-wave elastography and muscle thickness measured by ultrasonography before and 24 h after eccentric exercise (ECC), *N* = 14.

To induce DOMS, we used the ECC protocol established and validated in previous studies (Madeleine et al., 2006, 2011, 2018; Kawczynski et al., 2007, 2012, 2018). We observed an increase in soreness intensity 24 h after ECC in line with previous research (Madeleine et al., 2011, 2018), but no changes in MVC and ROM. The lack of changes in MVC and ROM can be explained by recruitment of additional motor unit, increased motor unit synchronization or altered contractile properties (Madeleine et al., 2011; Penailillo et al., 2015). In agreement with current findings, Nosaka et al. (2002) showed that DOMS appears to be independent of decline in MVC and ROM. Moreover, Yanagisawa et al. (2015) reported the absence of significant correlations between muscle stiffness evaluated by elastography and other exercise-induced muscle damage indicators, such as decreased joint ROM and muscle soreness. However, their results showed a significant increase in serum creatine kinase activity, suggesting that muscle damage did occur as a result of ECC. Similarly, Kawczynski et al. (2018) observed no decline in MVC of the UT muscle, despite a significant decrease in muscle belly stiffness and an increase in soreness intensity. In addition, we showed spatial heterogeneity in UT muscle dynamic stiffness, elastic modulus and thickness depicted in the 3D topographical maps (Figure 3) underlining the relevance of a bioengineering approach to map theROI (Binderup et al., 2010a). We observed a consistent spatial distribution from before to 24 h after ECC for all three parameters, i.e., the UT dynamic stiffness remained highest for point 4 (most distal) after ECC, while elastic modulus showed highest values in more proximal regions (point 2). As suggested in previous studies (Kelly et al., 2018; Madeleine et al., 2018), point 2 location corresponds to a thicker muscle belly site, where the SWE technique provides measurements from deeper tissue structures. The most distal point (point 4) is located closer to a superficial musculotendinous site, where stiffness of superficial structures can be assessed with myotonometry (Kawczynski et al., 2018). The spatial heterogeneity may be explained by differences in local structural features such as pennation angles, and fiber type (Damon et al., 2008). As suggested by other authors, – the spatial heterogeneity in elastic modulus could also result from non-uniform muscle damage induced by ECC (Green et al., 2012) or heterogeneous activation of muscle fibers during exercise (Kinugasa et al., 2006). Furthermore, Alfuraih et al. (2018) confirmed that the depth of measurement may influence the SWE elastic modulus. They showed that variability of the measurements increases quadratically with the depth of acquisition. Due to the anatomy of UT muscle, the depth of measurements could differ across the four points of data acquisition causing spatial heterogeneity in UT elastic modulus.

### Strengths and Limitations

The selection of UT as the muscle of interest was based on the fact that this body region is greatly affected by DOMS and musculoskeletal pain (Madeleine et al., 2018). As suggested in previous research, monitoring of UT muscle stiffness is essential for the prevention of rotator cuff tendinopathy and other sport-related injuries (Leong et al., 2016). Therefore, our results add new important information for clinicians and applied researchers with a focus on injury prevention and rehabilitation. Showing how a single muscle adapts to ECC could also support training load programming. Conducting multiple, instead of a single, measurement for each point for SWE and myotonometry, allowed us to obtain high reliability of these measurements (Vain and Kums, 2002; Kelly et al., 2018). All ultrasound measurements were blinded prior analysis, which allowed us to avoid subjectivity bias. As reported by Andonian et al. (2016) measurements performed in a slack resting position are more reliable and easier to standardize. However, as showed by Huang et al. (2018), testing in a functional position could better reflect real situations. Therefore, performing our measurements only in a resting position should be considered as a first limitation of the current study. Secondly, we did not investigate the intra-operator and day-to-day reliability of myotonometry and SWE in measuring the UT muscle stiffness. We based our reliability on previous results from our research group (Kawczynski et al., 2018), and others (Andonian et al., 2016). Therefore, we acknowledge the need for further research including the test-retest reliability, coefficient of variation and typical error. Thirdly, we conducted our measurements only 24 h after ECC. Adding measurements immediately after ECC would inform about the specific effects of exercise on the results. Notably, Kawczynski et al. (2018) showed that UT muscle stiffness decreased by 6.5% immediately after ECC, and by 14.2% at 24 h after ECC. The small change in stiffness immediately after ECC suggests that exercise in itself had a small influence on the results at 24 h. Moreover, according to Alfuraih et al. (2017), using different ROI sizes, probe orientations and measurement locations produce variability of SWE estimation. These factors can be considered as limitations. However, as proved by the same authors, SWE demonstrates the strongest internal agreement when the probe is placed longitudinally to the muscle fibers, with ROI of medium-to-large size, excluding myotendinous or myoaponeurotic structures (Alfuraih et al., 2017), which is in line with our settings. Finally, due to differences in anatomy, the available area for calculating SWE was not always uniform across subjects and some variations may have occurred due to the dimensions of the ROI.

## Conclusion

This pilot study shows for the first time that biomechanical muscle properties, represented by elastic modulus and dynamic muscle stiffness, decreased 24 h after ECC. Moreover, we present a novel 3D topographical maps showing spatial heterogeneity of muscle dynamic stiffness, elastic modulus and thickness, which may provide insight about within-muscle changes following ECC. Findings from the present study demonstrate that monitoring UT muscle stiffness using both myotonometry and SWE contribute to the understanding of how a single muscle adapts to ECC.

## Data Availability Statement

The raw data supporting the conclusions of this article will be made available by the authors, without undue reservation.

## Ethics Statement

The studies involving human participants were reviewed and approved by the Research Ethics Committee of North Denmark (N-2016-0023). The study was conducted in accordance with the Declaration of Helsinki. Informed consent was obtained from each participant. The patients/participants provided their written informed consent to participate in this study.

## Author Contributions

AKi, PM, AKa, and RL planned the study. PM and RL carried out the phases of the protocol and supervised the stiffness and thickness measurements. AKa performed the statistical analysis. AKi collected the data and drafted the manuscript. ZI and BC supervised methods and manuscript preparation. All authors read and approved the final version of the manuscript.

## Conflict of Interest

The authors declare that the research was conducted in the absence of any commercial or financial relationships that could be construed as a potential conflict of interest.
